# Two Complete Mitogenomes of Chalcididae (Hymenoptera: Chalcidoidea): Genome Description and Phylogenetic Implications

**DOI:** 10.3390/insects12121049

**Published:** 2021-11-23

**Authors:** Huifeng Zhao, Ye Chen, Zitong Wang, Haifeng Chen, Yaoguang Qin

**Affiliations:** 1 Hebei Key Laboratory of Animal Diversity, College of Life Science, Langfang Normal University, Langfang 065000, China; zhaohf@lfnu.edu.cn (H.Z.); chenye@lfnu.edu.cn (Y.C.); chenhaifeng@lfnu.edu.cn (H.C.); 2 School of Forestry, Northeast Forestry University, Harbin 150040, China; wangzitong@nefu.edu.cn

**Keywords:** mitogenome, Chalcidoidea, Chalcididae, phylogeny

## Abstract

**Simple Summary:**

The Chalcididae are a moderate-sized family of the superfamily Chalcidoidea in Hymenoptera, comprising 1548 species in 87 genera worldwide. Some species are potential natural enemies of pests in agriculture and forestry. Currently, there are still some controversies about the monophyly of Chalcididae and the phylogenetic relationships between Chalcididae and other families in Chalcidoidea. Based on the fact that no mitogenomic phylogenetic analyses of all of the published mitogenomes of Chalcidoidea have been conducted and no complete mitogenome of Chalcididae species has been reported, two newly completed mitochondrial genomes of Chalcididae species (*Brachymeria lasus* and *Haltichella nipponensis*) were sequenced and analyzed. The results show that the two chalcidid mitogenomes have quite similar structures and features. In phylogenetic analyses based on 13 PCGs of mitogenomes, the basal position and monophyly of Chalcididae within Chalcidoidea were supported by all trees derived from maximum likelihood (ML) and Bayesian inference (BI) methods.

**Abstract:**

The complete mitochondrial genomes of two species of Chalcididae were newly sequenced: *Brachymeria lasus* and *Haltichella nipponensis*. Both circular mitogenomes are 15,147 and 15,334 bp in total length, respectively, including 13 protein-coding genes (PCGs), two ribosomal RNA genes (rRNAs), and 22 transfer RNA genes (tRNAs) and an A+T-rich region. The nucleotide composition indicated a strong A/T bias. All PCGs of *B. lasus* and *H. nipponensis* began with the start codon ATD, except for *B. lasus*, which had an abnormal initiation codon TTG in ND1. Most PCGs of the two mitogenomes are terminated by a codon of TAR, and the remaining PCGs by the incomplete stop codon T or TA (ATP6, COX3, and ND4 in both species, with an extra CYTB in *B. lasus*). Except for trnS1 and trnF, all tRNAs can be folded into a typical clover structure. Both mitogenomes had similar control regions, and two repeat units of 135 bp were found in *H. nipponensis*. Phylogenetic analyses based on two datasets (PCG123 and PCG12) covering Chalcididae and nine families of Chalcidoidea were conducted using two methods (maximum likelihood and Bayesian inference); all the results support Mymaridae as the sister group of the remaining Chalcidoidea, with Chalcididae as the next successive group. Only analyses of PCG123 generated similar topologies of Mymaridae + (Chalcididae + (Agaonidae + remaining Chalcidoidea)) and provided one relative stable clade as Eulophidae + (Torymidae + (Aphelinidae + Trichogrammatidae)). Our mitogenomic phylogenetic results share one important similarity with earlier molecular phylogenetic efforts: strong support for the monophyly of many families, but a largely unresolved or unstable “backbone” of relationships among families.

## 1. Introduction

The Chalcididae are a moderate-sized family of the superfamily Chalcidoidea in Hymenoptera, with about 87 genera and 1548 species worldwide. This family appears in greatest diversity in the tropics. Members of Chalcididae have a medium to large body size, which varies from 1.5 to 15 mm in length, and they represent some of the largest specimens in Chalcidoidea [1]. Most species of Chalcididae are solitary primary parasitoids of Lepidoptera, Diptera and Coleoptera, along with a few hyper-parasitizing tachinids or ichneumonids [2], and thus can regulate populations of other insects in the ecosystems naturally.

The monophyly and phylogenetic relationships of Chalcididae in Chalcidoidea have been controversial for a long time. Morphologically, the Chalcididae was treated as a monophyletic group based largely on four putative synapomorphies: labrum exposed and contiguous with anterior clypeal margin, base of mandibles exposed and straight, parascutal and axillar carinae meeting at the transscutal articulation, and genal carina [3,4]. However, the genal carina in Eurytomidae and Pteromalidae, and the highly homoplastic nature of the three other features in Chalcidoidea, make the monophyly of Chalcididae seem unreliable [3]. As for the phylogenetic relationships of the Chalcididae, they have often been considered to be closely related to Eurytomidae and Leucospidae in morphological phylogenetics [3].

Molecular efforts, devoted to reconstructing the phylogeny of Chalcidoidea, include analysis of ribosomal markers [5,6], and extensive transcriptomic data [7,8]. In the analysis of ribosomal genes (18S and 28S), five subfamilies of the Chalcididae are not monophyletic [6]. However, the combined morphological and molecular characteristics strongly support the monophyly of the Chalcididae [9]. Additionally, all these results generally suggest that the Chalcididae never cluster with Eurytomidae and Leucospidae [5,7,8].

These conflicting results cause the aforementioned phylogenetic question to remain controversial and indicate the requirement for using various molecular data to understand the systematic position and the monophyly of Chalcididae within Chalcidoidea. Mitogenome data seem a good choice to answer these questions. Mitogenomes of insects are circular DNA molecules that code for 13 proteins, 22 transfer RNA genes, and two ribosomal RNAs [10]. Mitogenome data have been widely used in phylogenetic analysis [11,12,13,14,15,16,17,18,19,20,21,22,23]. Until now, however, only one partial mitogenome of the Chalcididae has been submitted to Genbank, which has significantly impeded the unveiling of systematic confusions of Chalcididae.

Here, the two full mitogenomes of *B. lasus* and *H. nipponensis* [24,25] were newly sequenced and analyzed, which contributed to better understanding of the characteristics of the mitogenome of the Chalcididae. In addition, phylogenetic analyses including 53 published mitogenomes together with our *de novo* data, which represented 10 families of Chalcidoidea, were carried out to assess the systematic position and monophyly of Chalcididae, and to deeply understand the phylogeny of Chalcidoidea.

## 2. Materials and Methods

### 2.1. Sample Preparation and DNA Extraction

*B. lasus* and *H. nipponensis* were collected in Xishuangbanna, Yunnan Province, China on 29 April 2019 (21°53′37′ N, 101°16′15′ E), and on 11 May 2019 (21°53′44.76′ N, 101°16′39.88′ E), respectively. Total genomic DNA was extracted using a DNeasy Blood & Tissue kit (QIAGEN, Dusseldorf, Germany), according to the manufacturer’s instructions. DNA concentrations were measured using a DeNovix DS-11 Spectrophotometer, and its integrity was examined with agarose gel electrophoresis by 0.5× TBE (Tris base, Boric acid and EDTA) buffer with 4 volts/centimeter for 45 min.

### 2.2. High throughout Sequencing

The genomic DNA of two chalcidids was qualified for next generation sequencing and was fragmented to 350 bp by a Covaris S220 Focused Ultrasonicator (Covaris, MA, USA). The sequence libraries were constructed using TruSeq DNA LT Sample Preparation Kit (Illumina, Inc., San Diego, CA, USA). After repairing the blunt ends, adenylating 3′ ends and ligating adapters, the fragmented DNA was enriched. Then, both libraries were pooled and sequenced using an Illumina Hiseq X10 platform. The obtained raw reads were filtered by removing adaptor sequences, contamination, and low-quality reads.

### 2.3. Data Assemble and Annotation

The clean data were assembled with MitoZ v2.4 [26]. The assembled circular mitogenomes were reordered COX1 as a start gene with the script ‘Mitogenome_reorder.py’ [26]. The annotation of the two mitochondrial genomes was performed using MitoZ and the MITOS2 online server (http://mitos2.bioinf.uni-leipzig.de/index.py, (accessed on 30 June 2021)), and the secondary structures of tRNAs were plotted by the MITOS2 web server. Furthermore, both mitogenomes were corrected manually.

### 2.4. Statistics of the Chalcididae Mitochondrial Genomes

The nucleotide composition of the whole mitogenome, PCGs, 22 tRNAs, and 2 rRNAs and the relative synonymous codon usage (RSCU) of the PCGs of both chalcidid mitogenomes were calculated in MEGA 5 [27]. Nucleotide compositional skew was calculated according to the following formula: AT skew = [A − T]/[A + T], GC skew = [G − C]/[G + C]) [28].

### 2.5. Phylogenetic Analysis

To investigate the phylogeny of Chalcididae in Chalcidoidea, we reconstructed the family-level relationships within Chalcidoidea using two datasets of the 13 PCGs with two inference methods (BI and ML). The mitogenomic phylogeny of Chalcidoidea was reconstructed with 53 ingroups (51 online data and 2 newly produced data in this study), representing 10 families, and 3 species close to Chalcidoidea were chosen as outgroups. The details of taxa are shown in Table 1.

The two datasets were PCG123 (13 PCGs including all codon positions) and PCG12 (13 PCGs without third codon positions). Bayesian inference (BI) and maximum likelihood (ML) methods were used to reconstruct phylogenetic trees. 

For PCG123 and PCG12 datasets, the best DNA model based on the Akaike information criterion (AIC) was obtained using jModeltest 2.1.7 [49] (Table S1), and those selected models were used by BI with the software MrBayes 3.2.6. To ensure that the average standard deviation of split frequencies was less than 0.01, eight million generations were run with sampling every 1000 generations. Node support was assessed by posterior probabilities (PPs). The ML analyses were performed using RAxML 8.2.4 [50] under the GTRCAT model, and branch support for the resulting phylogenies was evaluated using 1000 bootstrap replicates (BS) with a partitioned strategy, and other settings were default.

Tracer v1.6 [51] was used to check the likelihoods of all parameters of BI analyses of the two datasets to ensure the effective sample size (ESS) values greater than 200. The consensus tree was calculated by discarding the first 25% trees. To verify the consistencies of the topologies, both BI and ML analyses were repeated two times, and the phylogenetic trees were visualized by Figtree v.1.4.3 [52].

## 3. Results and Discussion

### 3.1. Mitogenome Organization and Base Composition

**Figure 1:**
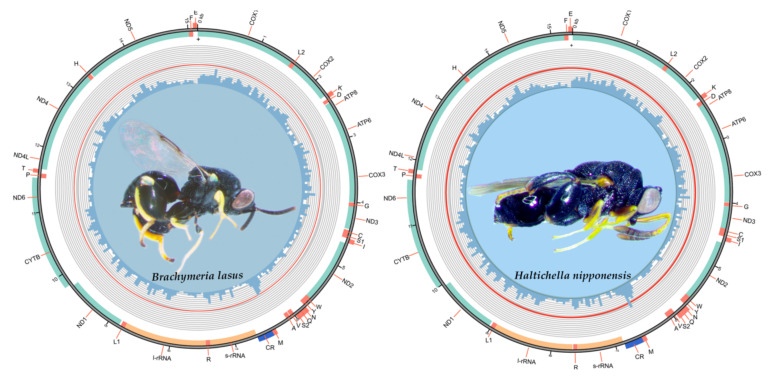
Complete mitochondrial genomes of *B. lasus* and *H. nipponensis*. The inner circle indicates the GC content in every 50-site window, and the outer circle shows the arrangement of the genes: light green for the PCGs, salmon for tRNAs, orange for rRNAs, and blue for control region.

The total lengths of mitogenomes in *B. lasus* and *H. nipponensis* are 15,147 bp and 15,334 bp, respectively. The both complete mitogenomes were investigated here, and were found to be composed of circular double-stranded molecules. Each mitogenome contains the typical set of 37 genes, including 13 PCGs, 22 tRNAs, 2 rRNAs and an A + T-rich area. The majority strand (J-strand) encodes 27 genes (11 PCGs, 14 tRNAs and 2 rRNAs), while the remaining 10 genes are located on the minority strand (N-strand) (two PCGs and eight tRNAs) (Figure 1, Table 2). The circular maps of the two mitogenomes are shown in Figure 1, and the details of annotations for the two complete mitogenomes are shown in Table 2.

In comparison to this newly sequenced complete mitogenome of *B. lasus*, the previous partial mitogenome of *Brachymeria* sp. is 15,092 bp in length [18], which contains two trnMs and lacks trnA and s-rRNA. The mitogenome map of *Brachymeria* sp. is shown in Figure S1. Another differentiation is the position of trnR. In *B. latus*, trnR is located between s-rRNA and l-rRNA while between trnQ and trnS2 in *Brachymeria* sp. Given no change of gene order between *B. lasus* and *H. nipponensis*, this result only suggests the existence of gene rearrangement in the genus *Brachymeria*.

Nucleotide composition for the two newly generated mitogenomes is shown in Supplementary Table S2. The entire sequence indicates a strong A and T bias: 84.5% for *B. lasus* and 83.9% for *H. nipponensis*. Excluding the A + T-rich regions, the highest AT content was found in the tRNA region, and the lowest was observed in the PCG region. Both of the whole mitogenomes show slightly negative AT-skews (−0.07 in *B. lasus* and −0.08 in *H. nipponensis*) and positive GC-skews (0.17 in *B. lasus* and 0.19 in *H. nipponensis*).

### 3.2. Protein-Coding Genes and Codon Usage

The total lengths of the 13 PCGs are 11,115 bp in *B. lasus* and 11,068 bp in *H. nipponensis*. The lengths of each PCG ranges from 156 bp (ATP8) to 1668 bp (ND5) in *B. lasus* and from 153 bp (ATP8) to 1665 bp (ND5) in *H. nipponensis*.

**Figure 2:**
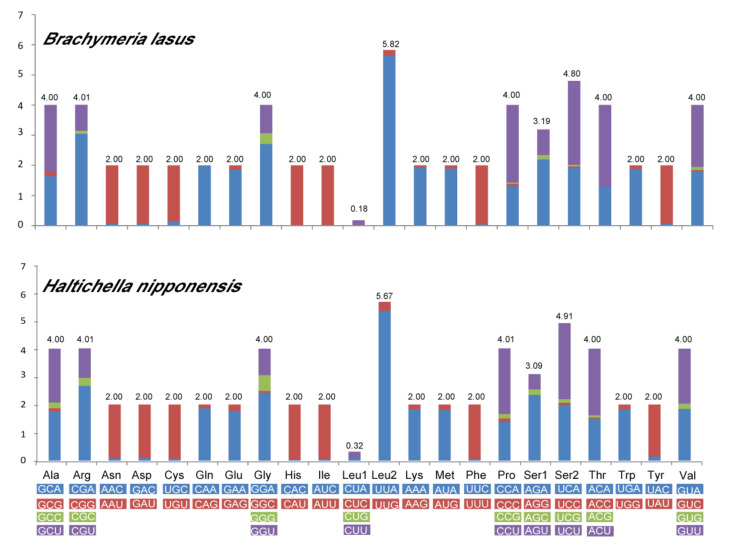
Relative synonymous codon usage (RSCU) in the PCGs of the new sequenced chalcidid mitogenomes. Codon families are indicated below the X axis.

The two mitogenomes of Chalcididae exhibited similar start and stop codons (Table 2). All the initiation codons of PCGs were ATD (ATA, ATG and ATT), except for ND1, which started with TTG in *B. lasus*, and ATT and ATG were the most frequently used. Three stop codons existed on the two new mitogenomic sequences: TAA, TA and a single T, and TAA was the most frequently used. Truncated termination codons are commonly used in metazoan mitogenomes, which could be completed by the post-transcriptional poly-adenylation [53]. The RSCU values of the two chalcids are shown in Figure 2. The codon UUA (Leu2) was the most commonly used in both mitogenomes.

### 3.3. Transfer and Ribosomal RNA Genes

**Figure 3:**
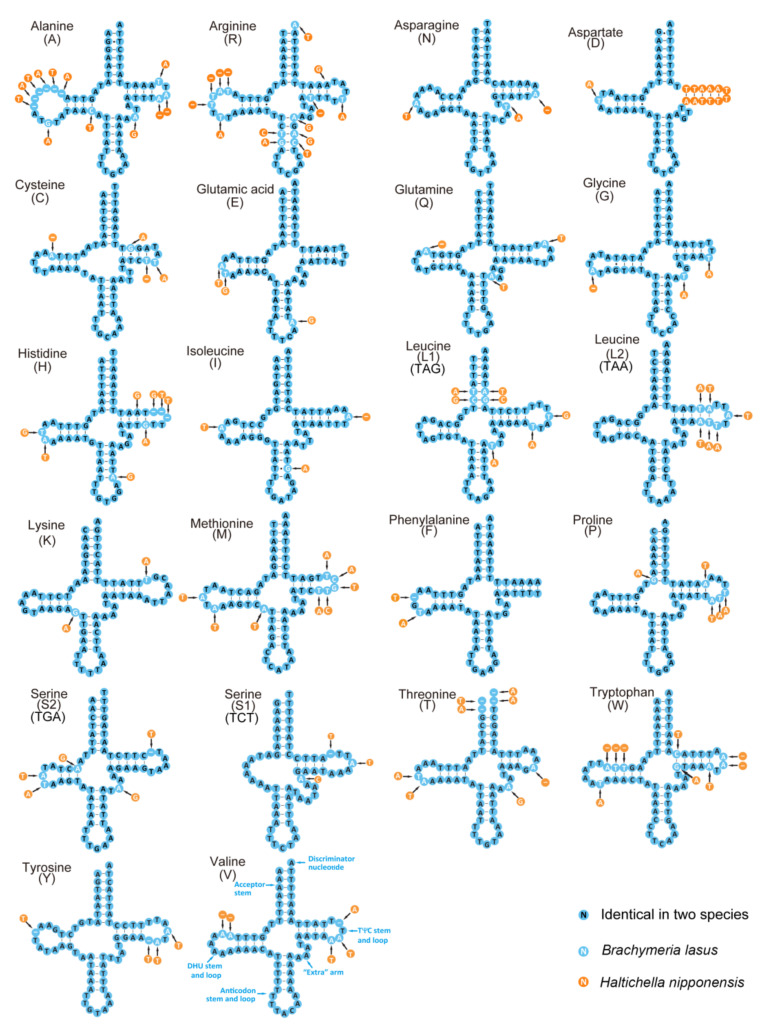
Predicted secondary structure for the tRNAs of *B. lasus* and *H. nipponensis*. The tRNAs are labeled with the abbreviations of their corresponding amino acids. Dashes indicate the Watson–Crick base pairs, and dots indicate the wobble GT pairs.

The secondary structures of the 22 tRNAs of the two Chalcididae species are shown in Figure 3. Both species possess the same entire length of tRNAs (1428 bp). The length of the 22 tRNAs ranged from 53 to 70 bp (Table 2). Most of the tRNAs could be folded into a typical clover-leaf structure, except for trnS1, which lost a dihydrouridine (DHU) arm, and trnF, which lost a TψC loop in the two species; furthermore, trnD lost a TψC arm in *B. lasus* (Figure 3). The secondary structures, comprised of the anticodon loop (7 nt) and anticodon stem (5 bp), are conserved in length, while the length of the acceptor stem (5–7 bp), DHU stem (3–4 bp, except for trnS1), and TψC stem (3–5 bp, except for the trnD in *B. lasus*) are variable. Additionally, the identified unmatched base pairs (GT) in different stems of tRNAs are shown in Figure 3, and these mismatched nucleotides might be restored during the post-transcriptional editing processes [54].

As for the rRNAs of the two species, both of l-rRNA (rrnL) and s-rRNA (rrnS) genes are encoded on the J-strand. The rrnL has a length of 1294 bp in *B. lasus* and 1265 bp in *H. nipponensis*, while rrnS has lengths of 731 and 728 bp. Both rRNAs have a heavy AT nucleotide bias, which reaches 86.7% and 85.8%, respectively. Similarly, a positive AT-skew and GC-skew are shown in the rRNAs of these two newly sequenced mitochondrial genomes.

### 3.4. A + T-Rich Region

In the mitogenome, the largest non-coding region is normally the A + T-rich region (also called the control region). The A + T-rich regions of Chalcididae mitogenomes are located between the rrnS and trnM genes, and the length was 236 bp for *B. lasus* and 289 bp for *H. nipponensis*. The A+T% content was 94.9% and 90.3% in the mitochondrial genomes of *B. lasus* and *H. nipponensis*, respectively. Though the alignment indicates that *B. lasus* and *H. nipponensis* share a similar control region, only in *H. nipponensis* were two repeat units of 135 bp found.

### 3.5. Phylogenetic Analysis

**Figure 4:**
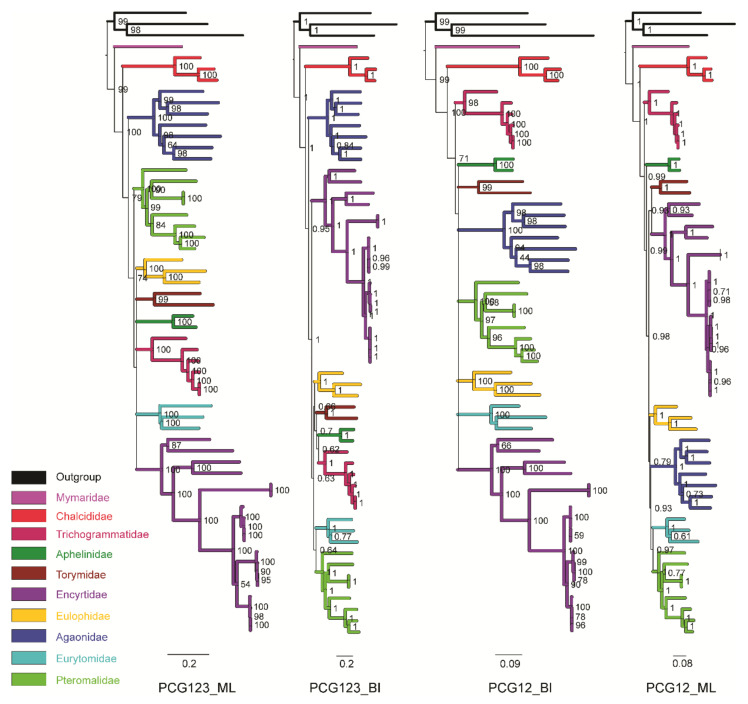
Phylogenetic trees constructed by ML/BI methods based on the dataset of PCG123 and PCG12. In the ML tree, all nodes of bootstrap value lower than 50 were shown as polytomy.

Phylogenetic analyses of two concatenated datasets (PCG123 and PCG12) were conducted using BI and ML, and are shown in Figure 4 (Supplementary Figures S2–S4). All the resulting trees supported the monophyly of Chalcididae, consistent with the previous comments derived from combined morphological and molecular characters [9], although this study only included two of five recognized subfamilies of Chalcididae. However, the result of ribosomal genes (18S and 28S) in Munro et al. [6] suggested that five subfamilies were scattered across the phylogenetic tree of superfamily Chalcidoidea. *Brachymeria* and *Haltichella* belong to the subfamilies Chalcidinae and Haltichellinae of Chalcididae, respectively. In our results, the phylogenetic relationship of *B. latus* was closer to *Haltichella*, with higher support values than *B*. sp. [18] (Supplementary Figures S2–S4). This result indicates that *Brachymeria* is paraphyletic, and needs validation by further studies. These issues alerted us the necessity of continuously sampling mitogenomes of other subfamilies in the future.

For the phylogenetic relationships in Chalcidoidea, all the resulting trees supported a hypothesis with a grouping of Mymaridae + (Chalcididae + remaining Chalcidoidea in our dataset), while the topologies between BI and ML trees showed apparent inconsistencies in the remaining Chalcidoidea. The basal position of Mymaridae was concordant with the published molecular results [5,6,7,8,9,55,56]. Chalcididae has been supported as the sister lineage with the remaining taxa of Chalcidoidea, excluding Mymaridae.

For the remaining Chalcidoidea, both the BI and ML results of the PCG123 dataset supported Agaonidae as the sister group of the other families with the medium support value (BS = 79; PP = 0.95). PCG123 trees also showed a similar topology to other families, except for Pteromalidae and Encyrtidae, and ML analysis supported Pteromalidae (BS = 74) as the sister group of these follow-up families, while BI supported Encyrtidae (PP = 1). Excluding Mymaridae and Chalcididae, the PCG12 dataset supported Trichogrammatidae as the sister group to other families with a high nodal value (BS = 71; PP = 0.99). Aphelinidae, as the next successive group, only received a high support value in BI analysis (PP = 0.99) (Figure 4; Supplementary Figures S2–S4).

All the trees seemed to share one important similarity with earlier molecular phylogenetic efforts: strong support for the monophyly of many families, but a largely unresolved or unstable “backbone” of relationships among families [5,6,7,8,9,55,56]. The Chalcidoidea are one of the most megadiverse groups of insects [2], whose family numbers appear to have undergone extremely rapid radiation in the post-Cretaceous era according to the fossil records and molecular dating hypothesis [7]. Therefore, resolving the phylogenetic relationships within radiated Chalcidoidea seems to be an extremely hard task.

## 4. Conclusions

In this study, two newly complete mitogenomes (*B. lasus* and *H. nipponensis*) have been sequenced and exhibited quite similar features in the genome size, base content, AT nucleotide bias, AT skew, GC skew, codon usage of protein genes, and secondary structure of tRNAs.

Phylogenetic analysis based on two datasets (PCG123 and PCG12) with two methods (maximum likelihood and Bayesian inference) indicated the monophyly of Chalcididae, although the sampling needs to be increased, and *Brachymeria*, as the largest genus in Chalcididae, might be not monophyletic. Our trees supported the basal position of Mymaridae, and recovered Mymaridae as the sister group of the remaining Chalcidoidea, as well as Chalcididae is the sister to the remaining chalcidoids, except for Mymaridae, in our mitogenomic phylogenetic analysis.

More mitogenomic data for Chalcididae and Chalcidoidea should be added to verify the monophyly of Chalcididae and elucidate the relationships between Chalcididae and other families in this mega-radiated superfamily in the future.

## Figures and Tables

**Table 1:** Mitogenomes of Chalcidoidea and outgroups used in this study.

Family	Taxa	GenBank Accession No.	References
Agaonidae	*Ceratosolen solmsi*	JF816396	[29]
	*Ceratosolen fusciceps*	MT916179	[30]
Agaonidae	*Dolichoris vasculosae*	MT947596	[31]
	*Eupristina koningsbergeri*	MT947597	[30]
	*Kradibia gibbosae*	MT947598	[30]
	*Wiebesia pumilae*	MT947601	[30]
	*Platyscapa corneri*	MT947604	[30]
Aphelinidae	*Encarsia formosa*	MG813797	[32]
	*Encarsia obtusiclava*	MG813798	[32]
Chalcididae	*Brachymeria* sp.	MG923487	[18]
	*Brachymeria lasus*	MZ615567	This study
	*Haltichella nipponensis*	MZ615568	This study
Encyrtidae	*Encyrtus infelix*	MH574908	[33]
	*Encyrtus infelix*	MH729198	[33]
	*Encyrtus sasakii*	MK111647	unpublished
	*Encyrtus sasakii*	MK111648	unpublished
	*Encyrtus sasakii*	MK189126	unpublished
	*Encyrtus sasakii*	MK189127	unpublished
	*Encyrtus eulecaniumiae*	MK189128	unpublished
	*Encyrtus eulecaniumiae*	MK189129	unpublished
	*Encyrtus eulecaniumiae*	MK189130	unpublished
	*Encyrtus eulecaniumiae*	MK189131	unpublished
	*Encyrtus rhodococcusiae*	MK189132	unpublished
	*Encyrtus rhodococcusiae*	MK189133	unpublished
	*Encyrtus rhodococcusiae*	MK189134	unpublished
	*Encyrtus rhodococcusiae*	MK189135	unpublished
	*Aenasius arizonensis*	MK630013	[34]
	*Diaphorencyrtus aligarhensis*	MN274569	[35]
	*Platencyrtus parkeri*	MN296710	unpublished
	*Metaphycus eriococci*	MW255970	unpublished
Eulophidae	*Tamarixia radiata*	MN123622	[31]
	*Necremnus tutae*	MT916846	[36]
	*Chouioia cunea*	MW192646	[37]
Eurytomidae	*Eurytoma* sp.	KX066374	[38]
	*Eurytoma* sp.	MG923494	[18]
	*Sycophila* sp.	MT947603	[30]
Mymaridae	*Gonatocerus* sp.	MF776883	[39]
Pteromalidae	*Philotrypesis* sp.	JF808722	[40]
	*Philotrypesis pilosa*	JF808723	[40]
	*Pteromalus puparum*	MG923513	[18]
Pteromalidae	*Pteromalus puparum*	MH051556	[41]
	*Pachyneuron aphidis*	MK577639	[42]
	*Apocrypta bakeri*	MT906648	[30]
	*Philotrypesis tridentata*	MT947602	[30]
	*Anisopteromalus calandrae*	MW817149	[43]
Torymidae	*Podagrion* sp.	MF795597	[44]
	*Torymus* sp.	MG923516	[18]
Trichogrammatidae	*Megaphragma amalphitanum*	KT373787	[1]
	*Trichogramma japonicum*	KU577436	[45]
	*Trichogramma ostriniae*	KU577437	[45]
	*Trichogramma dendrolimi*	KU836507	unpublished
	*Trichogramma chilonis*	MT712144	unpublished
	*Trichogramma chilonis*	MW789210	unpublished
Cynipoidea	*Trichagalma acutissimae*	MN928529	[46]
Platygastroidea	*Telenomus remus*	MT906647	[47]
Proctotrupoidea	*Trichopria drosophilae*	MN966974	[48]

**Table 2:** Features of the mitogenomes of *B. lasus* (left) and *H. nipponensis* (right).

Feature	Strand	Position (from)	Position (to)	Length	Intergenic Nucleotides	Anticodon	Initial Codon	Stop Codon
*COX1*	J	1/1	1542/1536	1542/1536	−5/−5		ATG/ATA	TAA
*trnL2*	J	1538/1532	1603/1597	66/66	0/0	TAA		
*COX2*	J	1604/1598	2269/2260	666/663	−8/−8		ATT	TAA
*trnK*	N	2262/2253	2331/2322	70/70	−1/−1	TTT		
*trnD*	J	2331/2322	2383/2385	53/64	13/0	GTC		
*ATP8*	J	2397/2386	2552/2541	156/155	−7/−7		ATT	TAA
*ATP6*	J	2546/2535	3218/3207	673/673	0/0		ATG	T
*COX3*	J	3219/3208	3999/3988	781/781	6/6		ATG	T
*trnG*	J	4006/3995	4070/4059	65/65	−3/−4	TCC		
*ND3*	J	4068/4056	4418/4406	351/351	18/−2		ATA	TAA
*trnC*	J	4437/4405	4501/4467	65/63	0/0	GCA		
*trnS1*	J	4502/4468	4560/4526	59/59	−1/0	TCT		
*trnI*	N	4560/4527	4626/4592	67/66	16/38	GAT		
*ND2*	J	4643/4631	5653/5617	1011/987	−2/−1		ATA/ATT	TAA
*trnW*	J	5652/5617	5720/5680	69/64	1/1	TCA		
*trnY*	J	5722/5682	5787/5749	66/68	1/1	GTA		
*trnN*	N	5789/5751	5854/5815	66/65	−1/−1	GTT		
*trnQ*	N	5854/5815	5921/5881	68/67	0/2	TTG		
*trnS2*	N	5922/5884	5986/5949	65/66	2/2	TGA		
*trnV*	J	5989/5952	6054/6016	66/65	10/0	TAC		
*trnA*	J	6065/6017	6128/6084	64/68	222/433	TGC		
*trnM*	N	6351/6518	6416/6583	66/66	0/0	CAT		
*CR*	J	6417/6584	6652/6872	236/289	0/0			
*s-rRNA*	J	6653/6873	7383/7600	731/728	−3/−4			
*trnR*	J	7381/7597	7446/7658	66/62	0/12	TCG		
*l-rRNA*	J	7447/7671	8740/8935	1264/1265	0/2			
*trnL1*	J	8741/8938	8808/9005	68/68	27/27	TAG		
*ND1*	J	8836/9033	9762/9959	927/927	44/42		TTG/ATT	TAA
*CYTB*	N	9807/10,002	10,959/11,156	1153/1155	−20/−20		ATG	T/TAA
*ND6*	N	10,940/11,137	11,521/11,688	582/552	1/31		ATT/ATG	TAA
*trnP*	J	11,523/11,720	11,590/11,784	68/65	6/3	TGG		
*trnT*	N	11,597/11,788	11,656/11,850	60/63	4/1	TGT		
*ND4L*	J	11,661/11,853	11,945/12,137	285/285	−7/−7		ATT	TAA
*ND4*	J	11,939/12,131	13,272/13,464	1334/1334	−1/−1		ATG	TA
*trnH*	J	13,272/13,464	13,333/13,528	62/65	13/12	GTG		
*ND5*	J	13,347/13,541	15,014/15,205	1668/1665	0/−1		ATT	TAA
*trnF*	J	15,015/15,205	15,078/15,269	64/65	−2/0	GAA		
*trnE*	N	15,077/15,270	15,141/15,334	65/65		TTC		

## Data Availability

Both sequences of mitogenomes were deposited in the GenBank under accession numbers of MZ615567 and MZ615568.

## Supplementary Materials

The following are available online at https://www.mdpi.com/article/10.3390/insects12121049/s1, Table S1: The best fit DNA model of each locus in datasets PCG123 and PCG12 selected by jModeltest 2.1.7, Table S2: Nucleotide composition of mitochondrial genomes of two Chalcididae species, Figure S1: The mitogenome map of *Brachymeria* sp., Figure S2: Phylogenetic relationships among Chalcidoidea species by BI analysis based on the PCG123 dataset, Figure S3: Phylogenetic relationships among Chalcidoidea species by ML analysis based on the PCG123 dataset, Figure S4: Phylogenetic relationships among Chalcidoidea species by BI analysis based on the PCG12 dataset, Figure S5: Phylogenetic relationships among Chalcidoidea species by ML analysis based on the PCG12 dataset.
